# Universal slow plasmons and giant field enhancement in atomically thin quasi-two-dimensional metals

**DOI:** 10.1038/s41467-020-14826-8

**Published:** 2020-02-21

**Authors:** Felipe H. da Jornada, Lede Xian, Angel Rubio, Steven G. Louie

**Affiliations:** 1 Department of Physics, University of California at Berkeley, Berkeley, CA 94720 USA; 2 Materials Sciences Division, Lawrence Berkeley National Laboratory, Berkeley, CA 94720 USA; 3 Max Planck Institute for the Structure and Dynamics of Matter and Center for Free-Electron Laser Science, Luruper Chaussee 149, 22761 Hamburg, Germany; 4 Center for Computational Quantum Physics, Flatiron Institute, New York, NY 10010 USA; 5 Present Address: Department of Materials Science and Engineering, Stanford University, Stanford, CA 94305 USA

**Keywords:** Two-dimensional materials, Electronic properties and materials

## Abstract

Plasmons depend strongly on dimensionality: while plasmons in three-dimensional systems start with finite energy at wavevector *q* = 0, plasmons in traditional two-dimensional (2D) electron gas disperse as $\omega _p \sim \sqrt q$. However, besides graphene, plasmons in real, atomically thin quasi-2D materials were heretofore not well understood. Here we show that the plasmons in real quasi-2D metals are qualitatively different, being virtually dispersionless for wavevectors of typical experimental interest. This stems from a broken continuous translational symmetry which leads to interband screening; so, dispersionless plasmons are a universal intrinsic phenomenon in quasi-2D metals. Moreover, our ab initio calculations reveal that plasmons of monolayer metallic transition metal dichalcogenides are tunable, long lived, able to sustain field intensity enhancement exceeding 10^7^, and localizable in real space (within ~20 nm) with little spreading over practical measurement time. This opens the possibility of tracking plasmon wave packets in real time for novel imaging techniques in atomically thin materials.

## Abstract (web summary)

Plasmons depend strongly on dimensionality. Here the authors show that plasmons in atomically thin metals are qualitatively different from those in a 2D electron gas or metal slab: they are dispersionless at large wavevectors and, in systems such as monolayer TaS_2_, long-lived enough to be observed experimentally as localized plasmon wave packets.

## Introduction

Plasmons are quantum collective motions of electrons in solids arising from the long-range Coulomb interaction. Since these propagating collective modes are strongly affected by boundary/geometric effects, plasmon modes can be tuned by assembling nanostructured materials with repeated patterns, or by selectively exciting modes that only exists on the surface of metals, such as the surface plasmon polaritons (SPPs)^1–4^. In addition, there has been great interest in plasmons in atomically thin (one to a few atomic layer) crystalline metals such as graphene^5–9^. Graphene plasmons are highly tunable and display the typical dispersion relation^10^ $\omega _p \sim \sqrt q$ of an ideal 2D electron gas for *q* → 0, and can be observed with techniques ranging from electron-energy loss spectroscopy (EELS) to nano-imaging of interference patterns generated with atomic-force microscopy (AFM) tips^1,2,4,11^.

Besides graphene, plasmons in other atomically thin quasi-2D metals have been reported^12–14^, some displaying intriguing dispersion relations. Previous ab initio studies have shown that plasmons on monolayer metallic transition metal dichalcogenides (TMDs) disperse as $\omega _p \sim \sqrt q$ at small *q* and can have high energy (~1 eV) that are nearly dispersionless for a large range of wavevectors (*q* ~ 0.1 Å^−1^ to 0.3 Å^−1^)^15–18^. Similar findings were found for other quasi-2D metals, such as borophene^19^. The previous studies however have basically overlooked the origin and significance of this dispersionless behavior. As will become clear in the present paper, dispersionless quasi-2D plasmons could find a variety of exciting applications, as flat dispersion relations can translate into real-space localization of plasmon wave packets using practical excitations. Moreover, it is important to unravel what is the physical origin of these dispersionless plasmons and to assess whether they are (or can be controllably made to be) sufficiently long lived for technological applications^20^.

**Fig. 1:**
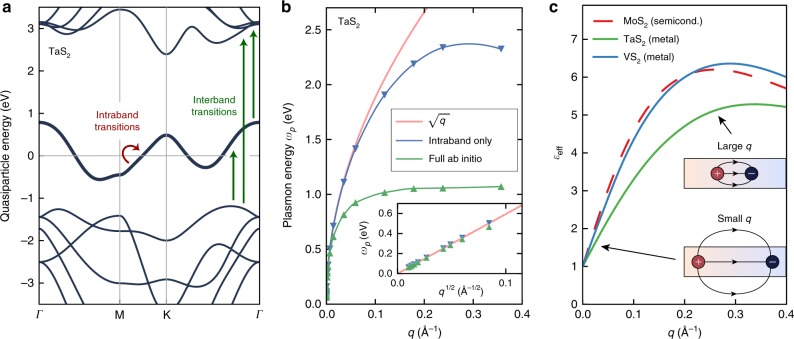
Plasmon dispersion relation of an atomically thin quasi-2D crystalline metal. **a** Calculated GW quasiparticle band structure of a prototypical quasi-2D metal, monolayer TaS_2_. The arrows highlight the intraband and interband electronic transitions that contribute to a plasmon excitation. The Fermi energy is set at zero. **b** Calculations of the plasmon dispersion relation of monolayer TaS_2_. The green curve is the result of the full ab initio calculation, which displays a flattening of the plasmon dispersion relation; the blue curve corresponds to the result of a calculation including only the intraband transitions, which displays a dispersion relation much closer to ~$\sqrt q$ (red curve). The inset shows the small-*q* behavior of the plasmon dispersion relation, where both calculations follow a $\sqrt q$ dependence. **c** Effective 2D dielectric function *ε*_eff_(**q**) for two point charges located at the innermost portion of three different quasi-2D materials. Since *ε*_eff_(**q**) does not include intraband (but only interband) transitions, its behavior is similar to quasi-2D semiconductors. For very small wavevectors, there is no screening (*ε*_eff_(**q** = **0**) = 1)) since very long wavelength perturbations correspond to charges being so far apart that they only experience the screening of vacuum. For increasing wavevector **q**, the charges get closer, so more of the field lines connecting them are confined to the interior of the quasi-2D material. *ε*_eff_(**q**) increases linearly with *q* until it reaches near a maximum, and subsequently decreases with increasing **q** after the maximum due to the intrinsic inability of the medium to screen very short-wavelength excitations.

Here, we demonstrate rigorously why the plasmon dispersion relation in real, quasi-2D metals is qualitatively different from that in idealized 2D metals (i.e., a homogeneous electron gas), in metallic slabs, and of the SPPs, tending to flatten at relatively large wavevectors for *any* quasi-2D crystals regardless of their chemical composition or nature of the energy bands crossing the Fermi energy. The local-fields screening arising from interband transitions in real quasi-2D crystalline metals^21–28^, which is not present in the 2D electron gas model, is the physics behind these dispersionless intrinsic plasmons. This behavior is universal for any atomically thin quasi-2D crystalline metal as long as there is a well-separated band (or set of bands) either completely occupied or unoccupied near the Fermi energy (as shown in Fig. 1a), a condition that is not fulfilled in doped graphene.

## Results

### Universal dispersionless plasmons in real quasi-2D metals

To shed light on this universal flat plasmon dispersion relation, we depict in Fig. 1 the computed plasmon dispersion relation from first principles for monolayer TaS_2_. To perform the calculation, with atomistic local-field effects, we have to develop an algorithm to compute plasmons for arbitrarily small wavevectors, which heretofore was a bottleneck for ab initio calculations (see Methods section). As expected from a homogeneous 2D electron gas picture, we find that plasmons in monolayer TaS_2_ in Fig. 1b follows a $\sqrt q$ dispersion relation up to a wavevector of *q* ~0.05 Å^−1^; however, the dispersion relation deviates significantly from this $\sqrt q$ relation afterwards (it becomes flattened), with an onset that is large compared to the wavevector of visible light but still very small compared to the Brillouin zone size of ~1.1 Å^−1^. This deviation is related to the broken continuous translational symmetry in real crystals (which only have discrete crystal translational symmetry). A homogeneous 2D electron gas with continuous in-plane translation symmetry can only have a single partially occupied electronic band that extends to infinite *q*, and only the intraband electronic transitions contribute to the system’s dielectric screening. On the other hand, both intraband and interband electronic transitions contribute to screening in a real quasi-2D metal such as monolayer TaS_2_, and, as we argue here, the interband transitions are the reason behind the appearance of virtually dispersionless plasmons. Indeed, the plasmon dispersion relation of monolayer TaS_2_ obtained by only considering intraband transitions (blue curve in Fig. 1b) follows a $\sqrt q$ dispersion relation up to much larger wavevectors^16^.

To gain some fundamental insights, we derive a general microscopic analytic expression for the plasmon dispersion relation for a quasi-2D metal, going beyond the 2D homogenous electron gas model. Our approach is to recast rigorously the plasmon excitations of a real quasi-2D material into contributions from two components: (1) an effective 2D electron gas (which is defined to consist of only those states of the system in the bands crossing the Fermi level); and (2) a nonlocal polarizable medium that incorporates the effects of the interband transitions in the real material (see Fig. 1a). Under the condition of long plasmon wavelengths, we derive (see SI section), by solving for the zeros of the total dielectric function, the following expression for the plasmon frequency,

1 $$\omega _p^2 = \frac{{2\pi e^2}}{{\varepsilon _{{\mathrm{eff}}}\left( {\mathbf{q}} \right)}}\left[ {\frac{n}{{m^ \ast }}\left( {\mathbf{q}} \right)} \right]_{{\mathrm{eff}}}q,$$

where the symbolic notation $\left[ {\frac{n}{{m^ \ast }}\left( {\mathbf{q}} \right)} \right]_{{\mathrm{eff}}}$ is a function that is a **q**-dependent generalization of the ratio of the carrier density *n* by the effective mass ${m^ \ast }$ of an anisotropic 2D system (see Methods section), which only depends on the nature of the band (or set of bands) crossing the Fermi energy. The plasmon frequency is further dependent on an effective 2D dielectric constant, *ε*_eff_(**q**), which includes contributions to the screening due to *all* the intrinsic interband transitions (indicated by the green arrows in Fig. 1a) of the quasi-2D material, excluding the intraband contributions from the set of bands crossing the Fermi energy, as well as due to any external environment (such as a substrate). As demonstrated below, *ε*_eff_(**q**) is responsible for the flat dispersion relation discussed above.

The fundamental idea here is that the effective dielectric function *ε*_eff_(**q**) from the interband transitions of the material essentially has the same analytical expression and behavior as the dielectric function derived in the context of quasi-2D semiconductor screening (see, e.g., Ref. ^28^), and it allows us to understand and determine, from Eq. (1), plasmon dispersion relations in quasi-2D metals using established models for quasi-2D semiconductors^21^ together with information from ab initio calculations^23,26–30^. In particular, the semiconductor-like interband effective 2D dielectric screening *ε*_eff_(**q**) of a monolayer material is not a constant, but increases linearly from unity with wavevector at small *q*, since very long-wavelength excitations effectively only experience screening from the vacuum or substrate (see Fig. 1c).

We show that, for long wavelength excitations, $\left[ {\frac{n}{{m^ \ast }}\left( {\mathbf{q}} \right)} \right]_{{\mathrm{eff}}}$ does not depend on *q*, while $\varepsilon _{{\mathrm{eff}}}\left( {\mathbf{q}} \right)$ can be approximated as $\varepsilon _{{\mathrm{eff}}}\left( q \right) \approx \frac{{1 + \varepsilon _s}}{2} + \rho _0q$, where *ε*_*s*_ is the dielectric constant of the insulating substrate and *ρ*_0_ is a screening length intrinsic to the quasi-2D material under consideration arising from the interband transitions (see Methods section). In this limit,

2 $$\omega _{\boldsymbol{p}} \approx \sqrt {2\pi {\mathrm{e}}^2\left[ {\frac{{\it{n}}}{{m^ \ast }}} \right]_{{\mathrm{eff}}}} \sqrt {\frac{{\it{q}}}{{\frac{{1 + \varepsilon _{\boldsymbol{s}}}}{2} + \rho _0{\boldsymbol{q}}}}} .$$

Equation (2) is one of the key results of this work. It shows that plasmons in real quasi-2D metals display a universal tendency to flatten for wavevectors above a critical wavevector *q*_*c*_ of the order of $\sim \frac{{\varepsilon _s + 1}}{{2\rho _0}}$, resulting in a plasmon frequency of $\omega _p^{{\mathrm{flat}}} = \sqrt {\frac{{2\pi {\mathrm{e}}^2}}{{\rho _0}}\left[ {\frac{{n}}{{{m}^ \ast }}} \right]_{{\mathrm{eff}}}}$ that is independent of the substrate dielectric constant *ε*_s_, although importantly the substrate dielectric constant can dramatically change the value of *q*_*c*_ and the dispersion relation for *q* < *q*_*c*_. We emphasize here that this phenomenon arises without considering interactions with photons, and hence is not a plasmon-polariton effect. Moreover, Eq. (2) holds in practice even for larger plasmon wavevectors, since the effective ratio $\left[ {\frac{n}{{m^ \ast }}} \right]_{{\mathrm{eff}}}\left( q \right)$ decreases for larger values of *q* due to a typical reduction of the quasiparticle band velocity away from the Fermi surface, while the effective interband screening *ε*_eff_(*q*) also saturates for larger *q*^28^. Since these two effects partially cancel each other, the main features predicted by Eq. (2)−that the plasmon dispersion relation flattens at larger *q* and has an asymptotic energy that does not depend on the substrate–holds from our ab initio calculations for all quasi-2D materials studied here up to *q* ~0.3 Å^−1^.

**Fig. 2:**
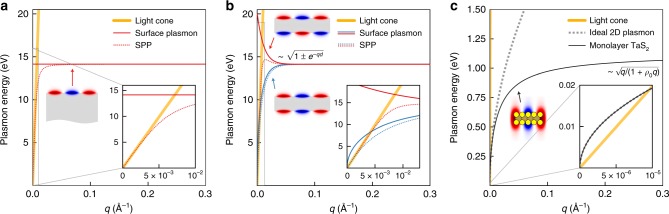
Comparison of plasmon excitations on the surface of a bulk metal, on a metallic thin film, and in atomically thin metals, which are different in nature. **a** Dispersion relation for a surface plasmon and the lower-energy surface plasmon-polariton (SPP) branch on the surface of a three-dimensional metal with a bulk plasmon energy $\hbar \omega _p^{{\mathrm{bulk}}}$ ~20 eV, similar to that of bulk TaS_2_. Retardation effects of the Coulomb interaction, which give rise to hybrid SPPs modes, are noticeable around the intersect of the light cone with the surface plasmon dispersion (inset). The colored intensity sketch represents the electric potential associated with the surface plasmon. **b** Same as **a**, but for a thin metallic film with thickness *d* = 100 $\AA$. The two distinct surface plasmon modes (red and blue solid lines), as well as the two lower-energy SPP modes (red and blue dashed lines), correspond to antisymmetric and symmetric relative oscillations of the electron density on the opposite surfaces, with the symmetric mode being lower in energy. **c** Dispersion relation for monolayer TaS_2_. The energy of the intrinsic plasmons in the dispersionless region is one order of magnitude smaller than that of the surface plasmons shown in **b**, and the critical wavevector 1/*ρ*_0 _≈ 0.04 ${\AA}^{ - 1}$ that defines the flat dispersion in monolayer TaS_2_ is about an order of magnitude smaller than that of a classical, atomically thin metallic film of the same thickness, which is 1/*d*_1L _= 0.2 ${\AA}^{ - 1}$ (*ρ*_0 _= 25 $\AA$ is the screening length and *d*_1L_ ~ 5 $\AA$ the thickness of monolayer TaS_2_). Moreover, when interaction with the light field is considered, the intersection of the light cone with the intrinsic plasmon dispersion in monolayer TaS_2_ occurs at a wavevector that is orders of magnitude smaller than the critical wavevector shown in Fig. 1b. Thus, the dispersionless plasmons in real quasi-2D materials that is the focus of this work are not the result of hybridizing a plasmon with a photon, i.e., it is not the same as the traditional plasmon polariton (inset).

The plasmon dispersion relation in atomically thin quasi-2D metals is in fact conceptually different from that of metallic slabs with thickness large compared to interatomic distance, which support on each surface (within the classical dielectric response framework) a surface plasmon-polariton dispersion relation that flattens at a wavevector $q_c \sim \frac{{\omega _p^{{\mathrm{bulk}}}}}{{\sqrt 2 c}}$, where $\hbar \omega _p^{{\mathrm{bulk}}}$ is the bulk plasmon energy^31,32^ (Fig. 2a). In contrast, as we demonstrated above, systems with atomically thin thickness support dispersionless quasi-2D plasmons with a very different *q*_*c*_ ~ 1/*ρ*_0_: the 2D screening length *ρ*_0_ in these systems (*ρ*_0_~25 Å for monolayer metallic TMDs) is not directly related to the thickness of the material nor the bulk plasmon energy^28^; it is a new length scale that emerges due to the quantum nature of these systems, which depends primarily on the energy for the interband transitions and the in-plane polarizability of the orbitals involved in these transitions. In addition, as mentioned above, neither retardation effects in the Coulomb interaction nor the hybridization of electronic transitions with the transverse electromagnetic fields are included in our calculations, so conventional hybrid plasmon-polariton modes cannot be responsible for the dispersionless plasmons in atomically thin metals. Further, the dispersionless quasi-2D plasmons here are also qualitatively and quantitatively different from plasmons in conventional thin metallic slabs, which support surface plasmons with an energy relation $\frac{{\hbar \omega _p^{{\mathrm{bulk}}}}}{{\sqrt 2 }}\sqrt {1 \pm e^{ - qd}}$, where the positive (negative) sign corresponds to a mode of antisymmetric (symmetric) relative oscillations of the electron density on the opposite surfaces, and *d* is the thickness of the slab^31,33,34^ (Fig. 2b). For materials such as bulk metallic TMDs, the bulk plasmon energy is of the order of 20 eV; a classical (and inappropriate) dielectric description of a metallic film of atomically thin thickness (*d* ~ 5Å) would yield a surface plasmon energy and a critical wavevector ~1/*d* for dispersionless surface plasmons that are both over one order of magnitude larger than the values found in our ab initio calculations for monolayer TaS_2_ (Fig. 2c). These differences highlight that the dispersionless plasmons in quasi-2D materials given by Eq. (1) are conceptually different from classical surface plasmons or hybrid plasmon-polariton modes, and they have instead their origin in the broken translational symmetry in atomically thin metals.

**Fig. 3:**
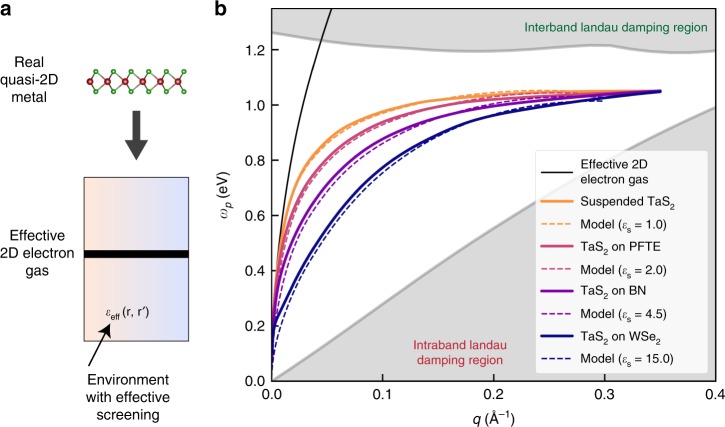
Ab initio results and model for plasmon dispersion relation of monolayer TaS_2_. **a** A real quasi-2D metal can be formulated as: a 2D electron gas with renormalized properties given by the energy band (or bands) that crosses the Fermi energy (thick band in Fig. 1a) embedded in an environment with an effective screening, which accounts for the interband transitions and environmental effects (Eq. (1) and Fig. 1c). **b** Calculated plasmon dispersion relation of monolayer TaS_2_ on a variety of substrates (Polytetrafluoroethylene (PTFE), BN, WSe_2_)–calculated from first principles (solid colored lines) and computed with the expression given in Eq. (1) (dash colored lines). For the latter, we fit the ab initio computed intrinsic effective dielectric screening *ε*_eff_(**q**) of suspended TaS_2_ to the form of a Keldysh model^21^. The resulting curves of the model on different substrates are then obtained without any additional fitting. The Landau damping regions are also shown (gray shaded regions), which is associated with the electron-hole continuum of either intraband or interband transitions (Fig. 1a).

We illustrate the accuracy of our expression in Eq. (1) by computing, from first principles^35,36^, the plasmon dispersion relation of monolayer TaS_2_ on a number of substrates spanning a range of *ε*_s_ ~ 2 to 10 (Fig. 3b). Our novel algorithm for fine sampling of transitions near E_F_ enables the ab initio calculation of the polarizability at arbitrarily small wave vectors **q** and without requiring mappings to model Hamiltonians, solving an important bottleneck in previous ab initio calculations (see Methods section). We compare our direct ab initio results to those from Eq. (1)–that is, by computing $\left[ {\frac{n}{{m^ \ast }}({\mathbf{q}})} \right]_{{\mathrm{eff}}}$ and *ε*_eff_(**q**) for a suspended monolayer TaS_2_, while generalizing *ε*_eff_(**q**) for the system on different substrates using the Keldysh model but based on ab initio parameters without any additional fitting (see Methods section). The excellent agreement in Fig. 3b confirms the idea that plasmons in quasi-2D metals can be understood as an effective 2D electron gas that experiences an additional screening (due to interband transitions and substrates) of a form of that is characteristic of quasi-2D semiconductors. Figure 3b also highlights that the plasmon group velocity can be changed by roughly an order of magnitude with a suitable choice of substrate, in addition to the tunable plasmon energies.

### Lifetime of quasi-2D plasmons

We now address the fundamental, and technologically important, question of what the lifetime of these collective excitations is. Plasmons are strongly damped when they are inside the Landau damping region, wherein they can decay into free electron-hole pairs^37^. Previous calculations^16^ on monolayer metallic TMDs employed density-functional theory (DFT) to compute the Landau damping regions and showed that plasmons in such systems coexist with single-particle excitations for *q* ≳ 0.15 Å^−1^ (that is, they are inside Landau damping regions for such large *q* plasmons), leading to a conclusion of short lifetimes. However, as static DFT is a ground-state theory, it does not yield the needed accurate quasiparticle energies to determine the correct, relevant Landau damping regions^35,36^ and hence plasmon lifetimes. By means of first-principles GW calculations for the quasiparticle excitations, we can accurately evaluate the Landau damping regimes in quasi-2D materials. Here, we focus on the monolayer metallic TMDs, as these materials are predicted (see Fig. 3) to have large plasmon energies^16,24^ and are intrinsically metallic and stable above their charge-density-wave^38^ and/or ferromagnetic^39^ transition temperature, if any. We find that, in these systems, DFT underestimates the separation between the half-filled *d* shell bands and the rest of the occupied bands by almost 200 meV and does not yield the correct threshold for single-particle interband excitations. Our quasiparticle GW calculations, on the contrary, show that the plasmon dispersion relation is well outside of the Landau damping regions, and coupling to finite-*q* interband transitions (electron-hole pair creations) are *not* a limiting factor for the plasmon lifetime for the materials studied here. The plasmons only reach the continuum of intraband transitions (thus having a decay channel to electron-hole pairs) after *q* ~ 0.4 Å^−1^ (Fig. 3b). Hence, monolayer metallic TMDs represent potential systems to implement the concept of lossless metals to support long-lived, dispersionless plasmons^40^. Other layered metals displaying a favorable electronic structure with interband transitions sufficiently separated in energy may also be good candidates^14^.

**Fig. 4:**
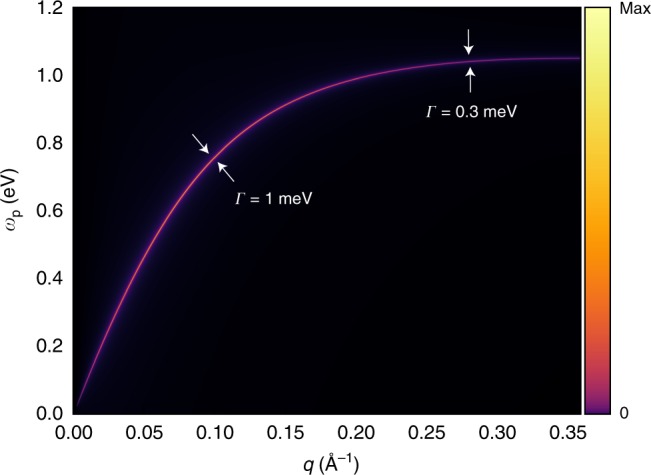
Plasmon spectral function with amplitude given in color scale for an intrinsic monolayer of TaS_2_ computed from first principles. We consider only decay processes solely from phonon-assisted scatterings, since the plasmon is outside of the Landau damping regions for the plotted range of wavevectors. The plasmon linewidth Γ becomes slightly sharper for modes with higher frequencies due to the smaller density of final states that the plasmon can decay to (see Methods section). The intrinsic plasmon linewidth decreases from 1 meV at ~0.15 $\AA^{{\mathrm{ - 1}}}$ down to 0.3 meV at *q* ~ 0.3 $\AA^{{\mathrm{ - 1}}}$.

Plasmons not in the Landau damping regions may also decay to free electron-hole pairs, through a higher order process, by additionally emitting or absorbing a phonon. While a first-principles calculation of this higher-order decay channel is quite involved, it can be estimated from a density of final states for decay, *D*(**q**), and an effective plasmon-phonon coupling matrix element $\bar g$ (see Methods section). We compute *D*(**q**) and estimate $\bar g$ for graphene and monolayer TaS_2_, both quantities from first principles. Even though *D*(**q**) is about two orders of magnitude larger for monolayer TaS_2_ than for graphene due to the larger band velocity in graphene, $\left| {\bar g} \right|^2$ is about 20 times larger for graphene due to the stronger chemical bonds. The product $D({\boldsymbol{q}})\overline g$, which determines the plasmon decay rate via phonons, is roughly the same for both systems. We plot in Fig. 4 the calculated **q**-resolved plasmon spectral function for monolayer TaS_2_. We obtain a plasmon lifetime *τ* ~ 2 ps from phonon-mediated decays, comparable to the lifetime of graphene plasmons measured in recent experiments^41^. More importantly, because plasmons in monolayer metallic TMDs originate mostly from electron orbitals of the innermost transition metal layer, we expect that the plasmon wavefunction does not extend too much outside the structure, and hence plasmons in monolayer metallic TMDs are more robust against substrate phonons than plasmons in graphene. While our estimate of the lifetime is an upper bound for an intrinsic sample, extrinsic effects such as ripples and defects, which needs to be considered in applications, can also be partially reduced with substrate engineering and encapsulation^42^.

### Slow plasmon wave packets

**Fig. 5:**
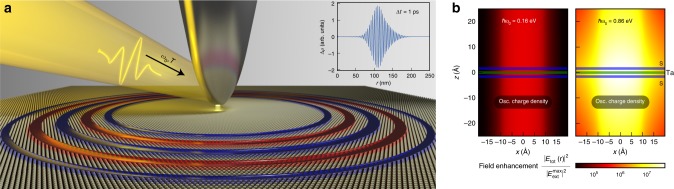
Slowly moving plasmon wave packet excited on monolayer TaS_2_. **a** Plasmon modes are excited in monolayer TaS_2_ with an ultrafast laser pulse (energy $\hbar \omega _0 = 1.02$ eV, modulated with a Gaussian profile of width of T = 80 fs), which is coupled to the sample through an AFM tip at ~4 Å above the topmost S atoms. The red and blue regions are schematic representations (not to scale) of the positively and negatively induced charge densities, respectively, that make up the plasmon wave packet at a time Δt = 1 ps after the external perturbation and are obtained from first principles. Inset shows a cross-sectional plot of the computed induced charge density which highlights that, 1 ps after the excitation, the plasmon wave packet only traveled ~100 nm and is still localized on a disk ~20 nm thick. **b** Field enhancement due to slow plasmons in monolayer TaS_2_. An external field is produced by an oscillating charge density, in the steady-state regime, with frequency *ω*_0_ distributed in a thin disk placed 10 Å below the bottom S layer. The field intensity enhancement, computed from first principles, is evaluated as the ratio of the intensity of the total electric field $\left| {E_{{\mathrm{tot}}}\left( {\mathbf{r}} \right)} \right|^2$ to the maximum intensity of the external field $\left| {E_{{\mathrm{ext}}}^{{\mathrm{max}}}} \right|^2$. The field intensity enhancement is two orders of magnitude larger and much more confined along the *z* direction perpendicular to the quasi-2D material when an external field with *ħω*_0_ = 0.86 eV excites slower and shorter-wavelength plasmons (right panel), compared to an external field with *ħω*_0_ = 0.16 eV which excites faster and longer-wavelength plasmons (left panel).

We now highlight how these finite-q, dispersionless quasi-2D plasmons can be used in experiments to create novel localized plasmon wave packets or use them for novel spectroscopic applications. We first consider a setup where plasmons are excited with an ultrafast laser pulse of duration *T* which is electromagnetically coupled to the sample through an AFM tip, commonly used to image plasmons^1,2,4,11^. We model this external excitation from the AFM tip as an oscillating point charge with time dependence $\rho _{{\mathrm{ext}}}\left( {{\mathrm{\Delta }}t} \right) \sim \sin \omega_0t \; e^{ - \frac{{{\mathrm{\Delta }}t^2}}{{2T^2}}}$ at ~4 Å above the topmost S atoms and compute the induced charge density as a function of space and time on a monolayer TaS_2_ for $\hbar \omega _0\sim 1$ eV and *T* ~ 80 fs. We find the resulting induced charge density moves very slowly through the material: after Δ*t* = 1 ps, the wave packet is still localized around a narrow (25 nm thick) ring with a radius of ~120 nm relative to the AFM tip (see inset of Fig. 5a). In addition, we can deduce that these plasmons are still well-defined excitations by computing the figure of merit *L*/*λ* ~ *v*_*g*_*τ*/*λ* ~ 60, where *L* is the plasmon propagation length and *λ* its wavelength, using the above discussed plasmon lifetime *τ* of ~2 ps and plasmon group velocity *v*_*g*_ ~ 0.6 Å/fs (see Methods section).

Slow plasmon wave packets excited such a way should allow one to use pump-probe timing information as a way to probe the electronic structure of complex heterostructures, since nano-imaging techniques employing AFM tips that image plasmon standing waves^1,2,4,11^ can be adapted to measure plasmon wave packets on quasi-2D metals. For instance, a slow plasmon wave packet created at time t_1_ can bounce back from the edge of the qusi-2D metal and eventually reach the AFM tip at time t_2_; the time difference t_2 _– t_1_ is a function not only of the distance the plasmon traveled, but also of the plasmon group velocity, which is very sensitive to the local screening environment (see Fig. 3b). Hence, compared to typical plasmon nano-imaging techniques, time-resolved plasmon microscopy should have a very high sensitivity to changes in the local and non-local electronic structure and can therefore detect changes in the substrate supporting a quasi-2D metal and the presence of point defects and interfaces even if these features are not directly on the monolayer material.

### Electric field enhancement

Nearly flat plasmon dispersion relations also allow for a high degree of confinement of the electromagnetic radiation: our first-principles calculations show that the resulting field intensity enhancement on both sides of a monolayer TaS_2_ is extraordinarily large−exceeding 10^7^ for an external field generated by a thin disk placed 10 Å under the bottom S layer (modeling a defect or nanostructured pattern) and oscillating with a time-dependent charge density at a frequency of $\hbar \omega _0 =$ 0.86 eV (see Fig. 5b and Methods section for details). This giant field intensity enhancement is indeed originating from the strongly localized and slowly moving plasmons. The enhancement obtained at a lower frequency of $\hbar \omega _0 =$ 0.16 eV, wherein the plasmon group velocity is larger by a factor of ~20×, is only of the order of 10^4^. But more importantly, as illustrated in Fig. 5, the field enhancement can be experienced in a wide spatial region on top of the monolayer material−only possible as the enhancement originates from the intrinsic quasi-2D plasmons of the 2D materials as opposed to from Mie scattering. This would allow for much easier accessing of these plasmon-induced field enhancements in experiments for biosensing, single-molecular spectroscopy, and catalytic applications, among others. Moreover, we envision that excitations of these slowly moving plasmons with light in larger metamaterials would open the possibility of creating arrays of coupled localized bosonic excitations in extended systems, enabling the monitoring of plasmon-plasmon interactions and scattering processes largely evasive to detailed and systematic studies up to now.

## Discussion

In summary, our ab initio calculations and effective model analysis show that plasmons in atomically thin quasi-2D metals follow a unique dispersion relation that flattens for larger wavevectors, owing to the characteristics of quasi-2D screening and broken translational symmetry in real atomically thin crystalline materials. We moreover show that monolayer metallic TMDs are especially suited to explore these nearly dispersionless plasmons, as they can support such collective excitations with long lifetime. The high tunability of these plasmons with varying substrates, as well as the flexibility to create plasmon wave packets with different group velocities, opens the possibility of exciting applications such as tracking plasmon wave packets in real time for time-resolved plasmonic imaging and supporting giant field enhancement (exceeding 10^7^) in extended quasi-2D metals over wide spatial regions.

## Methods

### New computational method for plasmon dispersion calculations

The key quantity required to obtain the plasmon dispersion is the proper, time-ordered polarizability matrix, which is given within the ring approximation by

$$\chi _{{\mathbf{GG}}^{\prime} }^0\left( {{\mathbf{q}},\omega } \right) = 	\frac{2}{{V_{{\mathrm{xtal}}}}}\mathop {\sum}\limits_{mn{\mathbf{k}}} \langle{n{\mathbf{k}}\left| {e^{ - i\left( {{\mathbf{q}} \,+\, {\mathbf{G}}} \right) \cdot {\mathbf{r}}}} \right|m{\mathbf{k}} + {\mathbf{q}}\rangle \langle m{\mathbf{k}} + {\mathbf{q}}\left| {e^{i\left( {{\mathbf{q}} \,+\, {\mathbf{G}}} \right) \cdot {\mathbf{r}}}} \right|n{\mathbf{k}}\rangle\,f_{m{\mathbf{k}} \,+\, {\mathbf{q}}}\left( {1-f_{n{\mathbf{k}}}} \right)} \\ \, 	\times \left[ {\frac{1}{{\omega - \left( {E_{n,{\mathbf{k}}} - E_{m,{\mathbf{k}} \,+\, {\mathbf{q}}}} \right) + i0^ + }} - \frac{1}{{\omega + \left( {E_{n,{\mathbf{k}}} - E_{m,{\mathbf{k}} \,+\, {\mathbf{q}}}} \right) - i0^ + }}} \right],$$

where *V*_xtal_ is the crystal volume, *m* and *n* denote band indices, **k** and **q** are wavevectors in the Brillouin zone, and *f* is a Fermi-Dirac occupation factor. The dielectric matrix *ε* is related, within the RPA, to the proper polarizability by $\varepsilon _{{\mathbf{GG}}^{\prime} }\left( {{\mathbf{q}},\omega } \right) = \delta _{{\mathbf{GG}}^{\prime} } - v\left( {{\mathbf{q}} + {\mathbf{G}}} \right)\chi _{{\mathbf{GG}}^{\prime} }^0\left( {{\mathbf{q}},\omega } \right)$, where *v*(**q** + **G**) is the Coulomb potential in reciprocal space and **G** is a reciprocal lattice vector.

In most ab initio codes, one typically needs to employ a uniform Monkhorst-Pack grid to sample all the **k** points for the virtual transitions involved in building *χ*^0^. Within this approach, the plasmon wavevector **q** is also typically made commensurate with the Monkhorst-Pack grid used to sample the **k** points. This leads to a serious computational challenge, as the number of **k** points *N*_*k*_ increases as $\left( {\frac{1}{q}} \right)^2$ for quasi-2D systems. Note that calculations on semiconductors do not exhibit this difficulty due to the presence of a gap. Here, we use an algorithm based on importance sampling to compute the polarizability matrix at arbitrarily small wavevectors, at a constant computational cost and with negligible and controllable error. First, we separate the proper polarizability matrix into intraband and interband contributions. The interband contribution, while may involve a large number of bands, can be easily sampled on a coarse **k**-point grid, since there is no Fermi surface to be resolved. The challenge is to efficiently sample the intraband contribution to *χ*^0^, which we denote by $\chi _{{\mathrm{intra}}}^0$.

In our new scheme to efficiently compute $\chi _{{\mathrm{intra}}}^0$, we sample only the set of **k** points that are involved with an intraband transition. We first distribute a set of special anchor points {**k**^*a*^} along the different pieces of the Fermi surface, in a way that: (i) **v**(**k**^*a*^)·**q** < 0, where **v**(**k**) is the band velocity at **k** (this will later ensure that the product of the occupation factors *f*_*m***k** + **q**_(1−*f*_*n***k**_) is nonzero); and (ii) their spacing along the direction perpendicular to **q** is fixed as 1/*N*_*a*_. Note that *N*_*a*_ is not the total number of anchor points, but the maximum number of anchor points that a closed and concave Fermi surface could have. We choose *N*_*a*_ to be large enough to finely sample transitions along the Fermi energy, around *N*_*a*_ ~ 60; however, note that *N*_*a*_ does not depend on the magnitude of *q*, but on the size of the Fermi surface. We then distribute the actual **k** points that will go into the calculation of $\chi _{{\mathrm{intra}}}^0$ in pairs around the anchor points, i.e., as $\left\{ {{\mathbf{k}}_i = {\mathbf{k}}_i^a \pm \frac{{\mathbf{q}}}{2}} \right\}_i$, so that each pair of **k** points will sample a transition along the various pieces of the Fermi surface.

We then perform the calculation of $\chi _{{\mathrm{intra}}}^0$ with these special set of **k** points and by including only intraband transitions. This is enforced by keeping only transitions between states $\left. {|m{\mathbf{k}} + {\mathbf{q}}} \right\rangle$ and $\left. {\left| {n{\mathbf{k}}} \right.} \right\rangle$ with a large overlap, $\left| {\langle {u_{m{\mathbf{k}} + {\mathbf{q}}}{\mathrm{|}}u_{n{\mathbf{k}}}}\rangle } \right|^2 \ge \frac{1}{2}$. We then compute interband transitions keeping only transitions such that $\left| {\langle {u_{m{\mathbf{k}} + {\mathbf{q}}}{\mathrm{|}}u_{n{\mathbf{k}}}}\rangle } \right|^2 < \frac{1}{2}$. Since these interband transitions do not sample the Fermi surface, they can be computed with two independent set of 6×6×1 **k**-point grids shifted by the plasmon wave vector **q**. Note that, both for the interband and intraband calculations, new set of wavefunctions need to be calculated depending on the wavevector **q**. The **k**-point grids can be chosen so that the **q**-dependent wavefunctions only depend on the valence states, so they can be efficiently recomputed. However, the approximation of only sampling the intraband transitions with set of pairs of **k**-points around the Fermi surface is only valid for small values of *q*. For values of *q* larger than 1/60 of the reciprocal lattice vector, we compute the polarizability with an uniform 60×60×1 **k**-point grid for transition involving well-separated occupied and unoccupied bands which are both ~2 eV from the Fermi energy, and employing a uniform 6×6×1 **k**-point for the remaining transitions. Altogether, we can accurately and efficiently compute the polarizability matrix from first principles for all relevant wavevectors.

**Fig. 6:**
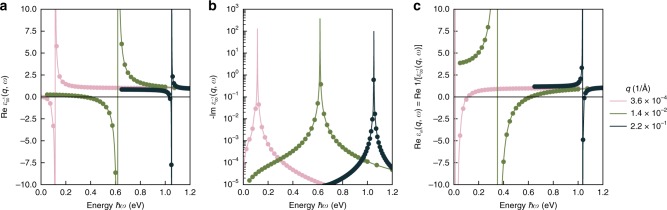
Dielectric response matrices computed from first principles for monolayer TaS_2_ in a supercell arrangement with a cell length of *L* = 100 Å along the direction normal to the layer and with numerical broadening of 0.1 meV. **a** Real and (**b**) imaginary parts of the **G** = **G**’ = 0 components of the inverse dielectric matrices of such dilutely stacked monolayers for three different in-plane wavevectors **q**, where **G** is a reciprocal lattice vector of the quasi-2D crystal. The imaginary part plotted in panel (**b**) is proportional to the loss function, the sharp peaks of which are associated with plasmon excitations. **c** Real part of the macroscopic dielectric matrix, the zeros of which are associated with plasmon collective excitations. While the absolute values of dielectric matrices depend on the stacking spacing and broadening parameter used in our supercell calculations, the poles in panels (**a**) and (**b**), as well as the zeros in panel (**c**), are not sensitive to such details.

Finally, we obtain the plasmon excitations by finding the peaks of the loss function $L\left( {{\mathbf{q}},\omega } \right) = - {\mathrm{Im}}\left[ {\frac{1}{{\varepsilon _m\left( {{\mathbf{q}},\omega } \right)}}} \right] = - {\mathrm{Im}}\, \varepsilon _{00}^{ - 1}\left( {{\mathbf{q}},\omega } \right)$, where $\varepsilon _m\left( {{\mathbf{q}},\omega } \right) = 1/\left[ {\varepsilon _{00}^{ - 1}({\mathbf{q}},\omega )} \right]$ is the macroscopic dielectric function. Finding the poles of ${\mathrm{Im}}\, \varepsilon _{00}^{ - 1}({\mathbf{q}},\omega )$ typically requires a very dense sampling of different frequencies *ω* around the plasmon. We avoid this by first decomposing $u({\mathbf{q}}) + iv({\mathbf{q}}) = 1/\varepsilon _{00}^{ - 1}({\mathbf{q}},\omega )$. Both *u***(q)** and *v***(q)** are smooth, real-valued functions near the peak of the loss function, so they can be accurately interpolated to give the loss function $L\left( {{\mathbf{q}},\omega } \right) = \frac{{v({\mathbf{q}})}}{{u({\mathbf{q}})^2 + v({\mathbf{q}})^2}}$, the maximum of which we associate with a plasmon energy *ω*_*p*_. The real and imaginary parts of the **G**=**G**’=0 components of the inverse dielectric matrix, as well as the real part of the macroscopic dielectric matrix, are shown in Fig. 6. While the absolute values of dielectric matrices reported are not directly observables (i.e., they depend on the stacking spacing used in our supercell calculations, as well as broadening parameters), the poles of the dielectric matrices are not sensitive to these details, and are associated with plasmon excitations.

### GW quasiparticle band structure

Our mean-field DFT calculations are performed with the Quantum ESPRESSO package^43^. We compute the quasiparticle band structure for monolayer TaS_2_ in the 2H phase, using the ab initio GW method^35^ with the BerkeleyGW package^36^, and using similar convergence parameters as we employed in previous studies on semiconducting monolayer TMDs^15^. A few notable differences are: (i) we perform the calculations without resorting to any plasmon-pole model by computing the fully frequency-dependent dielectric matrix and self-energy, (ii) we include all unoccupied bands in the calculation of the polarizability matrix and self-energy, and (iii) in order to sample a very dense **k**-point grid, we use the nonuniform neck subsampling (NNS) method^44^.

### Microscopic analysis and dispersion relation of quasi-2D plasmons

We identify plasmon collective excitations with peaks in the loss function $L\left( {{\mathbf{q}},\omega } \right) = - {\mathrm{Im}}\frac{1}{{\varepsilon _m\left( {{\mathbf{q}},\omega } \right)}} = - {\mathrm{Im}}\, \varepsilon _{00}^{ - 1}\left( {{\mathbf{q}},\omega } \right)$, where $\varepsilon _m({\mathbf{q}},\omega ) = \frac{1}{{\varepsilon _{00}^{ - 1}({\mathbf{q}},\omega )}}$ is the macroscopic dielectric function, and $\varepsilon _{00}^{ - 1}$ is the **G** = **G**’ = 0 component of the inverse dielectric matrix. The condition for a plasmon peak is that Re[det *ε*] = 0. Following the previous discussion, we can separate the proper polarizability into an interband contribution and an intraband contribution and assume for simplicity here that there is a single partially occupied band that is responsible for the intraband transitions.

The real part of the intraband contribution to the proper RPA polarizability, in the long wavelength limit and at zero temperature, is given by

$${\mathrm{Re}} \, \chi _{{\mathrm{intra}}}^0\left( {{\mathbf{r}},{\mathbf{r}}^{\prime} ,{\mathbf{q}},\omega } \right) =	 \frac{2}{{V_{{\mathrm{xtal}}}}}\mathop {\sum }\limits_k u_{\mathbf{k}}^ \ast \left( {\mathbf{r}} \right)u_{{\mathbf{k}} \,+\, {\mathbf{q}}}\left( {\mathbf{r}} \right)u_{{\mathbf{k}} \,+\, {\mathbf{q}}}^ \ast \left( {{\mathbf{r}}^{\prime} } \right)u_{\mathbf{k}}\left( {{\mathbf{r}}^\prime } \right)\theta ( {E_{{\mathbf{k}} \,+\, {\mathbf{q}}} - E_F})\theta \left( {E_F - E_{\mathbf{k}}} \right)\\ 	\times \left[ {\frac{1}{{\omega - ( {E_{{\mathbf{k}} + {\mathbf{q}}} - E_{\mathbf{k}}})}} - \frac{1}{{\omega + ( {E_{{\mathbf{k}} + {\mathbf{q}}} - E_{\mathbf{k}}})}}} \right],$$

where *u*_**k**_(**r**) is the periodic part of the Bloch function for the band crossing the Fermi energy with wavevector **k**, and *E*_*F*_ is the Fermi energy.

Since we are interested in plasmon solutions, we know that we evaluate $\chi _{{\mathrm{intra}}}^0$ at *ω* = *ω*_*p*_ ≫ *E*_**k** + **q**_ − *E*_**k**_, the reason being that $\omega _p \sim \sqrt q$ but *E*_**k** + **q**_ − *E*_**k**_ scales like **q** in the long wavelength limit. If we define $\rho _{\mathbf{k}}\left( {\mathbf{r}} \right) \equiv u_{\mathbf{k}}^ \ast \left( {\mathbf{r}} \right)u_{{\mathbf{k}} \,+\, {\mathbf{q}}}\left( {\mathbf{r}} \right)$ and assume that we are in the long wavelength limit, we can rewrite $\chi _{{\mathrm{intra}}}^0$ in a basis-set independent way for *ω* near *ω*_*p*_ as

$${\mathrm{Re}}\,\chi _{{\mathrm{intra}}}^0\left( {{\mathbf{q}},\omega \to \omega _p} \right) \approx \frac{{q^2\rho \rho ^T}}{{\omega ^2L_z}}\left[ {\frac{n}{{m^ \ast }}\left( {\mathbf{q}} \right)} \right]_{{\mathrm{eff}}},$$

where we define

$$\left[ {\frac{n}{{m^ \ast }}\left( {\mathbf{q}} \right)} \right]_{{\mathrm{eff}}} \equiv \frac{1}{{2\pi ^2}}\mathop {\int}\nolimits_{{\mathrm{FS}}} {{\mathrm{d}}k\,v_k\left( {\hat q \cdot \hat v_k} \right)^2} ,$$

*L*_z_ is the length of the simulation supercell along the normal direction of the plane of the quasi-2D material, FS denotes an integration along the Fermi surface, **v**_*k*_ is the band velocity for a wavevector at the Fermi surface, and $\left[ {\frac{n}{{m^ \ast }}\left( {\mathbf{q}} \right)} \right]_{{\mathrm{eff}}}$ is a **q**-dependent function that is a generalization of the ratio of the carrier density *n* to the effective mass ${m^ \ast }$.

The real part of dielectric matrix for frequencies near *ω*_*p*_ can now be written as

$${\mathrm{Re}}\,\varepsilon = I - v\left( {\mathbf{q}} \right)\chi ^0\left( {\mathbf{q}} \right) = \varepsilon _{{\mathrm{inter}}}\left( {\mathbf{q}} \right) - v\left( {\mathbf{q}} \right)\frac{{q^2\rho \rho ^T}}{{\omega ^2L_z}}\left[ {\frac{n}{{m^ \ast }}\left( {\mathbf{q}} \right)} \right]_{{\mathrm{eff}}},$$

where $\varepsilon _{{\mathrm{inter}}}\left( {\mathbf{q}} \right): = I - v\left( {\mathbf{q}} \right)\chi _{{\mathrm{inter}}}^0\left( {\mathbf{q}} \right)$ is the interband contribution to the dielectric matrix, which we may safely assume is purely real in the frequency range of interest. Using Sylvester’s determinant identity, the condition for a plasmon peak is equivalent to

$${\mathrm{Re}}[ {\det \varepsilon ( {{\mathbf{q}},\omega _p})}] 	= 1 - \frac{{q^2}}{{\omega_p ^2L_z}}\left[ {\frac{n}{{m^ \ast }}\left( {\mathbf{q}} \right)} \right]_{{\mathrm{eff}}}\rho ^TW_{{\mathrm{inter}}}\left( {\mathbf{q}} \right)\rho = 0\\ W_{{\mathrm{inter}}}\left( {\mathbf{q}} \right) 	\equiv \, \varepsilon _{{\mathrm{inter}}}^{ - 1}\left( {\mathbf{q}} \right)v\left( {\mathbf{q}} \right),$$

and *W*_inter_ is the Coulomb potential screened by the interband transitions.

In the long wavelength limit, if the charge density *ρ* is localized in the monolayer, we can approximate

$${\rho} ^TW_{{\mathrm{inter}}}\left( {\mathbf{q}} \right)\rho \approx {\mathop {\sum }\limits_{G_z,G^{\prime}_z}} \left[ {W_{{\mathrm{inter}}}\left( {\mathbf{q}} \right)} \right]_{G_z,G^{\prime}_z},$$

where *G*_*z*_ is a reciprocal-lattice vector along the confined direction of the material.

As done in the context of quasi-2D semiconductors^28^, we define an effective quasi-2D dielectric function as

$$\frac{1}{{\varepsilon _{{\mathrm{eff}}}({\mathbf{q}})}} = \frac{q}{{2\pi e^2L_z}}\mathop {\sum }\limits_{G_z,G^{\prime}_z} \left[ {W_{{\mathrm{inter}}}\left( {\mathbf{q}} \right)} \right]_{G_z,G^{\prime}_z},$$

and finally arrive at the expression for the plasmon frequency,

$$\omega _p^2 = \frac{{2\pi e^2}}{{\varepsilon _{{\mathrm{eff}}}\left( {\mathbf{q}} \right)}}\left[ {\frac{n}{{m^ \ast }}\left( {\mathbf{q}} \right)} \right]_{{\mathrm{eff}}}q.$$

### Comparison of first-principles calculations with analytical model

In order to assess the accuracy of the closed-form plasmon dispersion relation used in the model in Eq. (1), we need to compute separately both the interband effective dielectric function, *ε*_eff_(**q**), and the effective ratio $\left[ {\frac{n}{{m^ \ast }}\left( {\mathbf{q}} \right)} \right]_{{\mathrm{eff}}}$ for a suspended monolayer TaS_2_. We evaluate *ε*_eff_(**q**) from first principles using the expression in the previous Methods section, while the ratio $\left[ {\frac{n}{{m^ \ast }}\left( {\mathbf{q}} \right)} \right]_{{\mathrm{eff}}}$ is obtained by a numerical calculation of the plasmon dispersion relation including only intraband transitions also from first-principles. For *q* → 0, this is equivalent to the integral written in the previous Methods section, but it is easier to evaluate in practice.

Next, we obtain the substrate-dependent plasmon dispersion for monolayer TaS_2_. To do so, we first fit *ε*_eff_(**q**) obtained for a suspended monolayer TaS_2_ to the fully *q*-dependent expression for the Keldysh model^21^,

$$\varepsilon _{{\mathrm{eff}}}\left( q \right) = \frac{\varepsilon }{2}{\mathrm{sech}}\left( {\frac{{qd}}{2} + \eta _2} \right){\mathrm{sech}}\left( {\frac{{qd}}{2} + \eta _1} \right)\sinh \left( {qd + \eta _1 + \eta _2} \right),$$

where $\eta _1 = \frac{1}{2}\log (\varepsilon + \varepsilon _s) - \frac{1}{2}\log (\varepsilon - \varepsilon _s)$, $\eta _s = \frac{1}{2}\log (\varepsilon + 1) - \frac{1}{2}\log (\varepsilon - 1)$, *ε*_*s*_ is the substrate dielectric constant, and *ε* and *d* are effectively fitting parameters intrinsic to the quasi-2D material that were obtained from the suspended calculation (for instance, *d* does not in general correspond to the thickness of the quasi-2D material). Note that, for small *q* and large ε, this expression is commonly approximated as $\varepsilon _{{\mathrm{eff}}}\left( q \right) \approx \frac{{1 \,+\, \varepsilon _s}}{2} + \rho _0q$, where $\rho _0 \approx \frac{{d\varepsilon }}{2}$ whose value requires explicit first-principles calculation.

For TaS_2_, we obtain that a fit with *ε* = 5.52 and *d* = 10.3 Å reproduces the ab initio curve for *ε*_eff_(*q*) up to 0.4 Å^−1^. We then obtain the values of *ε*_eff_(*q*) for different substrates by varying *ε*_*s*_, which highlights the versatility of the model in Eq. (1). We take values of *ε*_*s*_ from experiment ($\varepsilon _s^{{\mathrm{PTFE}}} \sim 2$, $\varepsilon _s^{{\mathrm{hBN}}} \sim 4.5$), with the exception of WSe_2_, for which we compute $\varepsilon _s^{{\mathrm{WSe}}_2} \sim 15$ from first principles. The accuracy of the resulting close-form dispersion relations is depicted in Fig. 3b of the main text.

### Framework for estimating plasmon lifetime

We consider the decay rate of a plasmon with momentum **q** to an arbitrary final state *f* using Fermi’s golden rule,

$$\Gamma \left( {{\mathbf{q}} \to f} \right) = \pi \mathop {\sum}\limits_f {\left| {M\left( {{\mathbf{q}} \to f} \right)} \right|^2\delta \left( {E_f - \omega _p\left( {\mathbf{q}} \right)} \right)} ,$$

where *M* is a coupling matrix element, and here we use atomic Rydberg units $(\hbar = 2m_e = e^2/2 = 1)$.

We consider the case of the decay of a plasmon outside the Landau damping region, such as plasmons in monolayer TMDs (see Fig. 3b), so that a direct decay of a plasmon to an electron-hole pair cannot conserve both energy and momentum. Under these conditions, we need to consider decay processes that involve the emission of a phonon, i.e., in the low-temperature limit. There are three kinds of decay processes to consider that are first-order in the electron-phonon coupling matrix: (1) the direct decay of a plasmon to the electronic ground state by emission of a phonon, (2) the decay of a plasmon to another plasmon by emission of a phonon, and (3) the decay of a plasmon to an electron-hole pair plus a phonon. Process (1) is not relevant for plasmons close to the dispersionless regime, where $\hbar \omega _p\left( {\mathbf{q}} \right)\sim 1\,{\mathrm{eV}}$, since the phonon energy in monolayer TMDs is smaller by at least one order of magnitude. Process (2), while possible, has a small amplitude, since there is a tight constraint on momentum conservation because of the plasmon dispersion. Therefore, we focus on process (3), which could in principle be quite different between monolayer TMDs and graphene.

We label the final states as follows: the emitted phonon has a wavevector **Q**, branch index *λ* and frequency ${\mathrm{\Omega }}_\lambda ^{{\mathrm{ph}}}({\mathbf{Q}})$, while the final electron-hole pair consists of an electron with band index *c* and wavevector **k** + **q** − **Q**, and a hole with band index *v* and wavevector **k**, any combinations which satisfy wavevector conservation. We write the decay rate associated with this process as

$$\Gamma ({\mathbf{q}}) = \pi \mathop {\sum }\limits_{{\mathbf{Q}}\lambda } \mathop {\sum }\limits_{vc{\mathbf{k}}} \left| {M\left( {{\mathbf{q}} \to {\mathbf{Q}},\lambda ,v,c,{\mathbf{k}}} \right)} \right|^2\delta \left( {{\mathrm{\Omega }}_\lambda ^{{\mathrm{ph}}}\left( {\mathbf{Q}} \right) + {\it{\epsilon }}_{c{\mathbf{k}} \,+\, {\mathbf{q}} \,-\, {\mathbf{Q}}} - {\it{\epsilon }}_{v{\mathbf{k}}} - \omega _p\left( {\mathbf{q}} \right)} \right),$$

where ${\it{\epsilon }}$_*n***k**_ is the quasiparticle energy for an electron or hole in band *n* and wavevector **k**.

The coupling matrix element *M* between an initial plasmon state $\left. {|{\mathbf{q}}} \right\rangle$ and a final state $\left. {|f} \right\rangle$ mediated by the electron-phonon interaction Hamiltonian *H*_*ep*_ can be expressed as

$$M\left( {{\mathbf{q}} \to {\mathbf{Q}},\lambda ,v,c,{\mathbf{k}}} \right) = \langle {f| {H_{ep}}|{\mathbf{q}}} \rangle$$

with

$$H_{ep} = N_k^{ - \frac{1}{2}}\mathop {\sum }\limits_{n\,m\,{\mathbf{k}}\,{\mathbf{Q}}\,\lambda } g_{nm\lambda }\left( {{\mathbf{k}}, - {\mathbf{Q}}} \right)c_{m{\mathbf{k}} - {\mathbf{Q}}}^\dagger c_{n{\mathbf{k}}}\left( {a_{ - {\mathbf{Q}}\lambda } + a_{{\mathbf{Q}}\lambda }^\dagger } \right),$$

where *N*_*k*_ is the number of **k** points in the Brillouin zone, g_*nmλ*_(**k**, **Q**) is the electron-phonon matrix element, and *a*^†^ and *c*^†^ denote the creation operator for a phonon and an electron, respectively. We write the initial plasmon state within the Tamm-Dancoff approximation in the basis of free electron-hole pairs, $\left. {|{\mathbf{q}}} \right\rangle = \mathop {\sum }\limits_{v^{\prime} c^{\prime} {\mathbf{k}}^{\prime} } A_{v^{\prime} c^{\prime} {\mathbf{k}}^{\prime} }^{\mathbf{q}}|\left. {c^{\prime} {\mathbf{k}}^{\prime} + {\mathbf{q}},v^{\prime} {\mathbf{k}}^{\prime} } \right\rangle$, where $A_{v^{\prime} c^{\prime} {\mathbf{k}}^{\prime} }^{\mathbf{Q}}$ is an expansion coefficient and $\left. {\left| {c^{\prime} {\mathbf{k}}^\prime + {\mathbf{q}},v^\prime {\mathbf{k}}^\prime } \right.} \right\rangle$ denotes a free electron-hole pair of Bloch states excited above the ground state, and we write the final state as $\left. {\left| f \right.} \right\rangle = |\left. {c{\mathbf{k}} + {\mathbf{q}} - {\mathbf{Q}},v{\mathbf{k}},{\mathbf{Q}}\lambda } \right\rangle$ as a *free* electron-hole pair plus a phonon. The normalization of the initial plasmon state requires that $A^{\mathbf{q}} \sim N_k^{ - \frac{1}{2}}$, so that $M\left( {{\mathbf{q}} \to {\mathbf{Q}},\lambda ,v,c,{\mathbf{k}}} \right) \sim N_k^{ - 1}\left[ {g_{cc\lambda }\left( {{\mathbf{k}} + {\mathbf{q}}, - {\mathbf{Q}}} \right) - g_{vv\lambda }\left( {{\mathbf{k}}, - {\mathbf{Q}}} \right)} \right]$.

A fully first-principle evaluation of Γ for a real quasi-2D material with multiple bands is challenging, as it involves the plasmon wavefunction and the fully band-dependent and wavevector-dependent electron-phonon coupling matrix elements. At this point, to make the calculation trackable, we approximate all this dependence into an effective electron-phonon coupling matrix element $\bar g$, which is estimated from typical values of $\left| {g_{mn}\left( {{\mathbf{k}},{\mathbf{Q}}} \right)} \right|$ found for each material. This give us an order-of-magnitude estimate of the plasmon-phonon coupling matrix element. Note that this approximation is partially justified by the fact that we are dealing with metallic systems, as the electron-phonon coupling matrix elements are reasonably smooth and do not diverge for vanishing phonon wavevectors.

This also motivates us to define a momentum-integrated joint-density of states (MI-JDOS) as

$${\text{MI}}{\hbox{-}}{\text{JDOS}}\left( {\upomega} \right) = \frac{1}{{N_k^2}}\mathop {\sum }\limits_{vc{\mathbf{kk}}^\prime } \delta \left( {{\it{\epsilon }}_{c{\mathbf{k}}^\prime } - {\it{\epsilon }}_{v{\mathbf{k}}} - \omega } \right),$$

which is a measure of how many electron-hole pairs can be created with an energy ${\upomega}$, regardless of the momentum.

We then arrive at the following simplified expression to estimate the plasmon decay rate,

$${\mathrm{\Gamma }} \sim \pi \,\bar g^2\,{\text{MI}}{\hbox{-}}{\text{JDOS}}\left( {\omega \left( {\mathbf{q}} \right) - {\mathrm{\Omega }}^{{\mathrm{ph}}}} \right),$$

where Ω^ph^ is a typical phonon frequency associated with $\bar g^2$. For monolayer metallic TMDs, we simply set Ω^ph^ ≈ 0.

We compute from first principles the momentum-integrated JDOS using DFT and GW calculations and compute the electron-phonon matrix elements using density-functional perturbation-theory calculations for graphene and monolayer TaS_2_. The DFT and GW calculations are performed with the Quantum ESPRESSO^43^ package and the BerkeleyGW^36^ package, respectively. We find that the MI-JDOS decreases as a function of energy for monolayer materials for $\hbar \omega \,\gtrsim\, 0.3\,{\mathrm{eV}}$. At the dispersionless region, we find that the monolayer TMD ${\text{MI}}{\hbox{-}}{\text{JDOS}}\left( {\hbar {\upomega} = 1\,{\mathrm{eV}}} \right) \approx 0.1\,\left( {{\mathrm{eV}}} \right)^{ - 1}$ per unit of TaS_2_. For graphene doped at *E*_*F*_ = 0.5eV, on the other hand, we find that ${\text{MI}}{\hbox{-}}{\text{JDOS}}\left( {\hbar {\upomega} = 1\,{\mathrm{eV}}} \right) \approx 0.001\left( {{\mathrm{eV}}} \right)^{ - 1}$ per two carbon atoms.

We estimate $\bar g^2$ by computing the average of the electron-phonon coupling matrix elements over all possible momenta for states at the half-filled band. For monolayer TaS_2_, the values of $\left| {g_\lambda ({\mathbf{k}},{\mathbf{q}})} \right|^2$ range up to 0.035 eV^2^ with an average of ~0.0005 eV^2^, the latter of which we assign to $\bar g^2$. For doped graphene, the values of $\left| {g_\lambda ({\mathbf{k}},{\mathbf{q}})} \right|^2$ range up to 0.26 eV^2^ with an average of ~0.01 eV^2^. Using these results for $\bar g^2$ together with the above calculated MI-JDOS, we obtain the plasmon linewidth given in Fig. 4.

The value of the plasmon lifetime $\tau = (2\Gamma {\mathrm{/}}\hbar )^{ - 1} \sim 2\,{\mathrm{ps}}$ allows us to estimate the figure of merit *L*/*λ* = *v*_*g*_*τ*/*λ*, where *L* is the plasmon propagation length, *λ* is its wavelength, and *v*_*g*_ the group velocity. For the wave packets used in the simulations to produce Fig. 5, we have that *q* ~ 0.3 $\AA^{ - 1}$ and *v*_*g* _~ 0.6 Å/fs, so *L*/*λ* ~ 60. In fact, the plasmons should be well-defined excitations (i.e., *L*/*λ* ≥ 1) provided that their lifetimes are longer than ~35 fs.

### Electric field enhancement

We compute the electric field enhancement from first principles by computing the total electric field $E_{{\mathrm{tot}}}\left( {{\mathbf{q}} + {\mathbf{G}},\omega } \right) = \mathop {\sum }\nolimits_{{\mathbf{G}}^\prime } \varepsilon _{{\mathbf{G}},{\mathbf{G}}^\prime }^{ - 1}\left( {{\mathbf{q}},\omega } \right)E_{{\mathrm{ext}}}({\mathbf{q}} \,+ {\mathbf{G}}^\prime ,\omega )$ given an external longitudinal field *E*_ext_ . One challenge to compute the total electric field in real space and from first principles is that one needs to perform a Fourier transform of a very sharp function, which depends on the matrix *ε*^−1^(**q**, *ω*). However, the dielectric matrices are only evaluated for a discrete number of wavevectors. We address this by writing the interacting RPA polarizability matrix close to a plasmon state and for a fixed frequency *ω*_0_ in terms of a spectral representation,

$$\chi _{{\mathbf{G}},{\mathbf{G}}^\prime }\left( {{\mathbf{q}},\omega _0} \right) \approx \frac{{C_{{\mathbf{G}},{\mathbf{G}}^\prime }\left( {{\mathbf{q}}_0} \right)}}{{v_g}}\frac{1}{{q - q_0 - i\eta }},$$

where *q*_0_ is the plasmon wavevector corresponding to the frequency *ω*_0_, i.e., *ω*_*p*_(q_0_) = *ω*_0_, *v*_*g*_ is the plasmon group at the wavevector *q*_0_, *η* ≡ *τ*^−1^/*v*_*g*_, where *τ* is the plasmon lifetime (the results do not change for *τ* ranging from 1 to 10 ps), and *C*_**G,G′**_(**q**) is an Hermitian matrix that can be fit from the ab initio inverse dielectric matrix. Note that the field enhancement in real space, which depends on the Fourier transform of *χ*_**G,G′**_(**q**, *ω*_0_) over its spatial directions, gets enhanced for slower plasmons.

Because of the mismatch in their wavelengths, plasmons in quasi-2D extended materials will not directly couple to light, and thus require a nanostructure with a length scale comparable to that of the wavelength of the plasmon to be excited. This is modeled here as an infinitesimally thin disk with a radius of 10 Å. Because the total field still depends considerably on the geometry of this nanostructure, we define the external field as the longitudinal field created by this nanostructure, which is assumed to be generated by an oscillating charge density uniformly distributed over the disk. We compute the field intensity enhancement as the ratio of the intensity of the total electric field $\left| {E_{{\mathrm{tot}}}\left( {\mathbf{r}} \right)} \right|^2$ to the maximum intensity of the external field $\left| {E_{{\mathrm{ext}}}^{{\mathrm{max}}}} \right|^2$ generated by this nanostructure. This definition is likely a lower bound to the field enhancement $\left| {E_{{\mathrm{tot}}}\left( {\mathbf{r}} \right)} \right|^2/I_{{\mathrm{light}}}$ defined with respect to the intensity *I*_light_ of an external planewave light source, since the nanostructure can also be engineered to enhance the intensity $\left| {E_{{\mathrm{ext}}}^{{\mathrm{max}}}} \right|^2/I_{{\mathrm{light}}}$.

## Data Availability

The data that support the findings of this study are available from the corresponding authors upon reasonable request.

## Code availability

Ab initio calculations were performed with the BerkeleyGW software package^36^. The new algorithms proposed here and the post processing scripts that support the findings of this study are available from the corresponding authors upon reasonable request.
